# Supplementary material for: Association between Body Mass Index and Diabetes Mellitus Are Mediated through Endogenous Serum Sex Hormones among Menopause Transition Women: A Longitudinal Cohort Study
Source: Int J Environ Res Public Health. 2023 Jan 19;20(3):1831. doi: 10.3390/ijerph20031831 (PMC9914507; doi:10.3390/ijerph20031831)
Supplement: Supplementary file 1 [file ijerph-20-01831-s001.zip › ijerph-2126246-supplementary.pdf]

The calculation details about the total effect, direct effect and indirect effect on the excess relative risk (ERR) scale.

According to the reference(VanderWeele, 2013), under the counterfactual framework, Y_1 and Y_0 were the counterfactual outcome given the exposure levels ($X = 1$ and $X = 0$), the conditional total effect on risk ratio scale was defined as:

$$RR_c^{TE} = E[Y_1 \mid c]/E[Y_0 \mid c]$$

Where c stands for all the confounders. M_1 and M_0 were the counterfactual outcome given the exposure levels ($X = 1$ and $X = 0$), and the direct effect (DE) and total direct effect (TIE) risk ratio was defined as:

$$RR_c^{DE} = E\left[Y_{1M_0} \mid c\right]/E\left[Y_{0M_0} \mid c\right]$$

$$RR_c^{TIE} = E\left[Y_{1M_1} \mid c\right]/E\left[Y_{1M_0} \mid c\right]$$

So that,

$$RR_c^{TE} = RR_c^{TIE} \times RR_c^{DE}$$

VanderWeele and Vansteelandt (Vanderweele & Vansteelandt, 2010) proposed as a measure of the proportion mediated on the risk difference scale the measurement $\frac{RR_c^{DE}(RR_c^{TIE}-1)}{(RR_c^{TE}-1)}$.

$RR_c^{TE} - 1$ is the excess relative risk (ERR) for the total effect. Convert to the proportion mediated on ERR scale:

$$\frac{RR_c^{DE}(RR_c^{TIE}-1)}{(RR_c^{TE}-1)} = \frac{(RR_c^{TE}-1)-(RR_c^{DE}-1)}{(RR_c^{TE}-1)} = \frac{RR_c^{TIE}-1}{(RR_c^{TE}-1)}$$

The mediating effect based on ERR scale is additive.

Based on the generalized linear regression model:

$$\text{logit}\,(P(Y = 1 \mid a, m, c)) = \theta_0 + \theta_1 a + \theta_2 m + \theta_3 am + \theta_4' c$$
$$E[M \mid a, c] = \beta_0 + \beta_1 a + \beta_2' c$$

The coefficient is expressed as follows:

$$RR_c^{DE} = \exp\,[\{\theta_1 + \theta_3(\beta_0 + \beta_1 + \beta_2' c + \theta_2\sigma^2)\} + 0.5\theta_3^2\sigma^2]$$

$$RR_c^{TE} = \exp\,[\theta_1 + \theta_2\beta_1 + \theta_3(\beta_0 + \beta_1 + \beta_2' c + \theta_2\sigma^2)\} + 0.5\theta_3^2\sigma^2]$$

Indirect effect is as follows:

$RR_c^{TIE} = \exp[\theta_1 + \theta_2\beta_1 + \theta_3(\beta_0 + \beta_1 + \beta_2' c + \theta_2\sigma^2)\} + 0.5\theta_3^2\sigma^2] - \exp[\{\theta_1 + \theta_3(\beta_0 + \beta_2' c + \theta_2\sigma^2)\} + 0.5\theta_3^2\sigma^2]+1$

More detailed derivation details are available in eAppendix for "A three-way decomposition of a total effect into direct, indirect, and interactive effects".

Supplementary Material

Table S1. Odd Ratios and 95%CIs for the overweight/obesity on hyperglycemia, diabetes, and pre-diabetes risk

	Unadjusted OR (95%CI)	*P*	Adjusted OR (95%CI)	*P*
Hyperglycemia (N=145)				
Normal	Reference	-	Reference	-
Overweight/Obesity	4.66 (3.04, 7.42)	< 0.001	4.06 (2.52, 6.80)	< 0.001
New diabetes (N=114)				
Normal	Reference	-	Reference	-
Overweight/Obesity	5.54 (3.36, 9.69)	< 0.001	3.81 (2.26, 7.20)	< 0.001
New pre-diabetes (N=31)				
Normal	Reference	-	Reference	-
Overweight/Obesity	4.68 (1.92, 1.24)	0.001	2.79 (1.29, 6.70)	0.013

Unadjusted ORs mean the coefficients in the model without any adjustments of covariates. Adjusted ORs mean the coefficients in the model with adjustment of baseline age, race, education, physical activity, smoking regularly, and follow-up years.

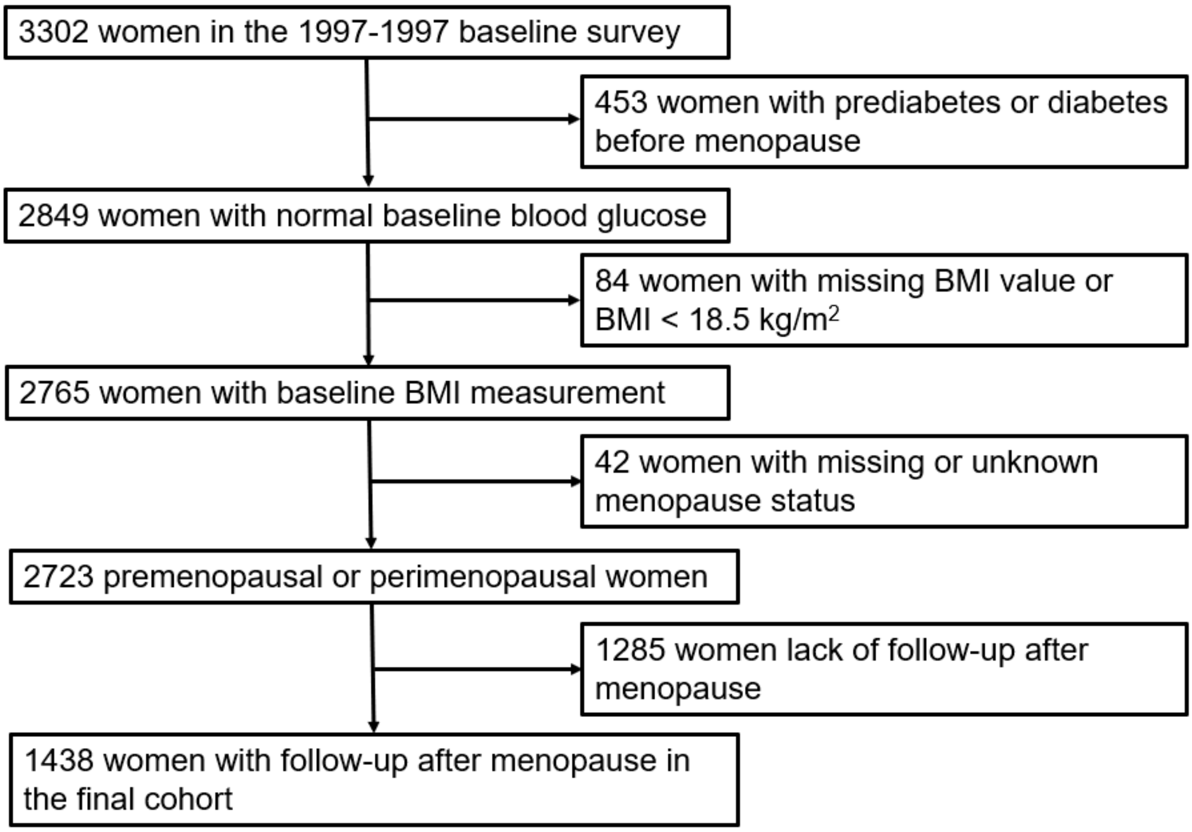

Figure S1. Inclusion and exclusion criteria.

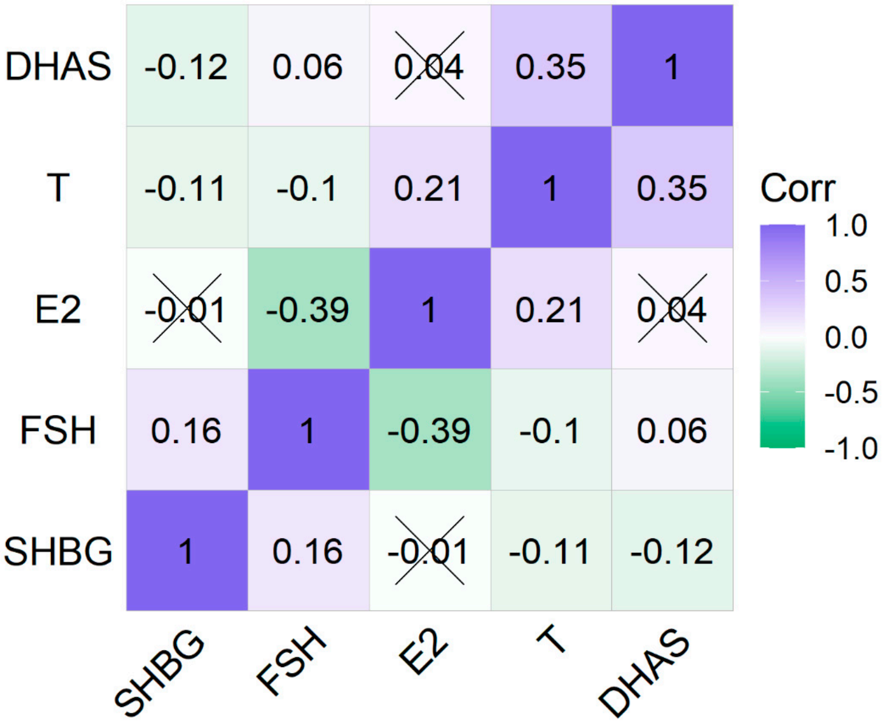

Figure S2. Correlation matrix between sex hormones. The symbol "×" indicates that the p-value of the correlation coefficient is higher than 0.05.

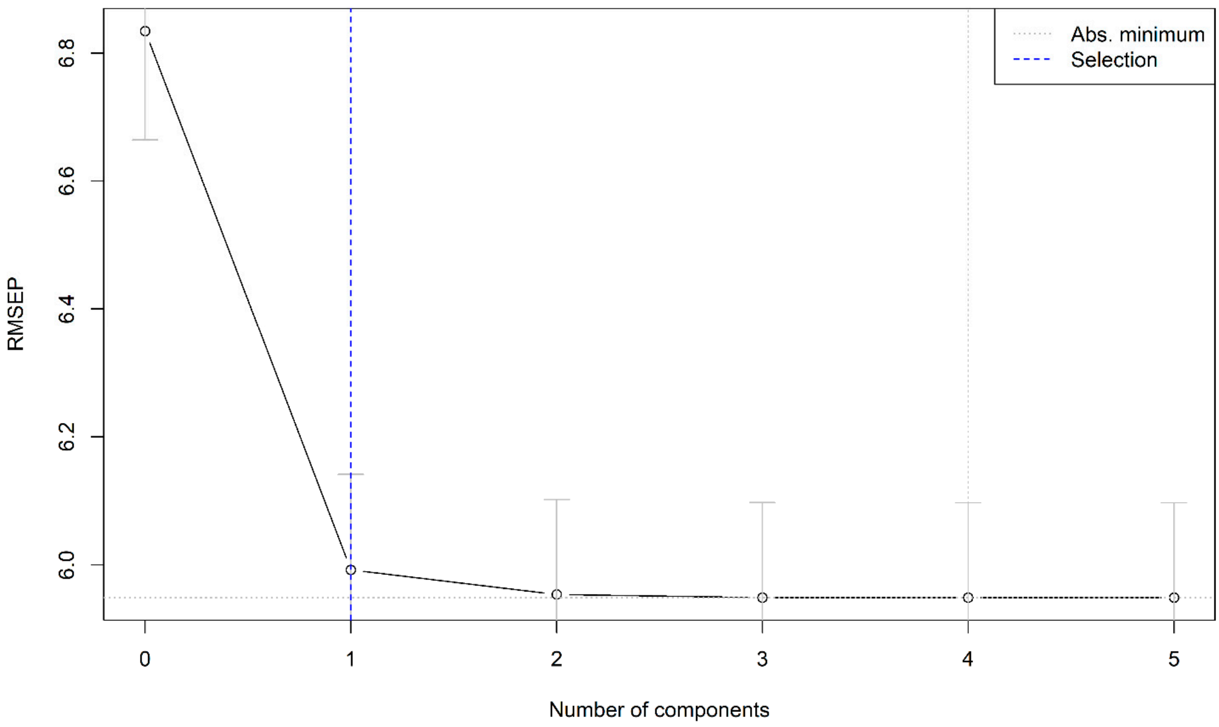

Figure S3. The number of components was selected by verification of root mean squared error of prediction (RMSEP).

Table S2. The Mediation effect of PLS hormone signal and each sex hormone on association between overweight/obesity and pre-diabetes or diabetes by excess relative risk (ERR) scale

	**New diabetes (N=114)**				**New pre-diabetes (N=31)**			
	NIE (95%CI)	NDE (95%CI)	TE (95%CI)	Mediation proportion%	NIE (95%CI)	NDE (95%CI)	TE (95%CI)	Mediation proportion%
PLS score	**1.38 (0.45, 2.31)***	2.49 (0.52, 4.45)*	3.88 (1.20, 6.55)*	**35.68 (21.83, 49.53)****	0.74 (-0.11, 1.60)	1.52 (-0.67, 3.72)	2.27 (-0.47, 5.00)	**32.67 (3.88, 61.47)***
SHBG	**0.88 (0.18, 1.58)***	3.17 (0.88, 5.46)*	4.05 (1.29, 6.84)*	**21.71 (9.99, 33.43)***	0.46 (-0.19, 1.12)	1.77 (-0.59, 4.13)	2.23 (-0.46, 4.93)	20.76 (-4.04, 45.57)
FSH	**0.87 (0.24, 1.49)***	2.96 (0.77, 5.15)*	3.82 (1.18, 6.47)*	**22.61 (11.99, 33.24)****	0.50 (-0.08, 1.09)	1.80 (-0.58, 4.18)	2.29 (-0.47, 5.06)	**21.70 (2.52, 40.89)***
T	-0.08 (-0.34, 0.18)	3.95 (1.25, 6.64)*	3.86 (1.22, 6.50)*	-2.13 (-8.72, 4.46)	0.09 (-0.23, 0.42)	2.14 (0.09, 3.36)	2.23 (-0.46, 4.93)	4.16 (-10.18, 18.52)
E2	0.22 (-0.16, 0.60)	3.64 (1.10, 6.19)*	3.87 (1.22, 6.51)*	5.72 (-3.69, 15.15)	-0.02 (-0.47, 0.41)	2.27 (-0.49, 5.02)	2.24 (-0.46, 4.95)	-1.27 (-21.08, 18.54)
DHAS	0.10 (-0.05, 0.26)	3.77 (1.18, 6.37)*	3.88 (1.23, 6.53)*	2.67 (-1.20, 6.55)	0.00 (-0.15, 0.16)	2.24 (-0.46, 4.94)	2.25 (0.46, 4.95)	0.16 (-6.71, 7.03)

SHBG, Sex Hormone Binding Globulin; T, Testosterone; E2, Estradiol; FSH, Follicle-stimulating hormone; DHAS, Dehydroepiandrosterone sulfate; PLS, Partial least squares analysis; NDE, Natural direct effect; NIE, Natural indirect effect. Mediation models were adjusted for baseline age, race/ethnicity, smoking regularly, education, physical activities and follow-up years.

*$P<0.05$, **$P<0.001$.

All coefficients were standardized by z-transformation before analysis.

Table S3. The Mediation effect of PLS score and each sex hormone on association between abdominal obesity and hyperglycemia by excess relative risk (ERR) scale

	NIE (95%CI)	NDE (95%CI)	TE (95%CI)	Mediation proportion%
PLS score	**1.60 (0.79, 2.40)****	2.46 (1.14, 3.79)**	4.06 (2.19, 5.93)**	**39.31 (26.93, 51.68)****
SHBG	**1.08 (0.48, 1.69)****	2.97 (1.50, 4.44)**	4.06 (2.20, 5.92)**	**26.75 (16.80, 36.71)****
FSH	**0.96 (0.41, 1.53)***	3.06 (1.54, 4.58)**	4.03 (2.18, 5.88)**	**24.07 (13.79, 34.35)****
T	-0.00 (-0.17, 0.17)	3.94 (2.14, 5.74)**	3.94 (2.15, 5.73)**	-0.07 (-4.43, 4.28)
E2	0.16 (-0.15, 0.48)	3.78 (2.02, 5.54)**	3.95 (2.15, 5.74)**	4.13 (-3.68, 11.95)
DHAS	0.01 (-0.21, 0.22)	3.93 (2.13, 5.73)**	3.94 (2.15, 5.73)**	0.21 (-5.25, 5.66)

SHBG, Sex Hormone Binding Globulin; T, Testosterone; E2, Estradiol; FSH, Follicle-stimulating hormone; DHAS, Dehydroepiandrosterone sulfate; PLS, Partial least squares analysis; NDE, Natural direct effect; NIE, Natural indirect effect; TE, Total effect. Mediation models were adjusted for baseline age, race/ethnicity, smoking regularly, education, physical activities and follow-up years.

*P<0.05, **P<0.001.

Abdominal obesity is defined as waist ≥ 85 cm. All coefficients were standardized by z-transformation before analysis.

Reference

1. VanderWeele, T.J. A Three-way Decomposition of a Total Effect into Direct, Indirect, and Interactive Effects. *Epidemiology* **2013**, *24*, 224–232. https://doi.org/10.1097/EDE.0b013e318281a64e.
2. Vanderweele, T.J.; Vansteelandt, S. Odds ratios for mediation analysis for a dichotomous outcome. *Am. J. Epidemiol.* **2010**, *172*, 1339–1348. https://doi.org/10.1093/aje/kwq332.
